# Nursing students’ perspectives on patients' safety competencies: a cross-sectional survey

**DOI:** 10.1186/s12912-024-01966-1

**Published:** 2024-05-13

**Authors:** Yasmin Ibrahim Abdelkader Khider, Shaimaa Mohamed Elghareeb Allam, Mohamed A. Zoromba, Heba Mohammed Mahmoud Elhapashy

**Affiliations:** 1 Medical-Surgical Nursing Department, Faculty of Nursing, Mansoura University, Mansoura, Egypt; 2 College of Nursing, Prince Sattam Bin Abdulaziz University, Al-Kharj, Saudi Arabia; 3 Psychiatric and Mental Health Nursing Department, Faculty of Nursing, Mansoura University, Mansoura, Egypt

**Keywords:** Nursing students, Patients’ safety, Competencies

## Abstract

**Background:**

Nurses constitute the largest body of healthcare professionals globally, positioning them at the forefront of enhancing patient safety. Despite their crucial role, there is a notable gap in the literature regarding the comprehension and competency of nursing students in patient safety within Egypt. This gap underscores the urgent need for research to explore how nursing students perceive patient safety and the extent to which these competencies are integrated into their clinical and educational experiences. Understanding these perspectives is essential for developing targeted interventions that can significantly improve patient safety outcomes. The objective of this study was to fill this gap by assessing the perspectives of nursing intern students on patient safety competencies, thereby contributing to the global efforts in enhancing patient safety education and practice.

**Methods:**

In this research, a cross-sectional study design was employed to investigate the topic at hand. A purposive sample of 266 nursing intern students was enrolled from the Faculty of Nursing at Mansoura University. The data were collected using a patient safety survey. Subsequently, the collected data underwent analysis through the application of descriptive and inferential statistical techniques using SPSS-20 software.

**Results:**

Among the studied intern nursing students, we found that 55.3% and 59.4% of the involved students agreed that they could understand the concept of patient safety and the burden of medical errors. Regarding clinical safety issues, 51.1% and 54.9% of the participating students agreed that they felt confident in what they had learned about identifying patients correctly and avoiding surgical errors, respectively. Concerning error reporting issues, 40.2% and 37.2% of the involved students agreed that they were aware of error reports and enumerated the barriers to incident reporting, respectively. There was a statistically significant difference between the nursing student patient safety overview domain and their age (*p* = 0.025).

**Conclusions:**

Our study's compelling data demonstrated that intern students who took part in the patient safety survey scored higher overall in all patient safety-related categories. However, problems with error reporting showed the lowest percentage. The intern students would benefit from additional educational and training workshops to increase their perspectives on patients' safety competencies.

**Supplementary Information:**

The online version contains supplementary material available at 10.1186/s12912-024-01966-1.

## Introduction

Patient safety refers to the perspectives, beliefs, attitudes, and values shared among members of the health community that focus on the prevention of errors and adverse effects on patients associated with health care [1]. In addition to becoming more efficient, health care has also grown more complicated due to the increased use of novel tools, medications, and therapies [2]. Medical errors (MEs) are a major public health concern that endangers patient safety significantly. Research conducted in Australia found that 16.6% of all admissions resulted in preventable negative outcomes, with approximately 5% of cases involving an iatrogenic injury ending in death [3].

Adverse event incidence rates varied from 2.9% to 16.6%. About 5% to 13% of the patients in these situations passed away, but 25% to 50% of them were thought to have been avoidable [4]. MEs can occur in any care setting, including hospitals, health centers, clinics, and laboratories; thus, they can negatively affect patient safety [5].

Medical errors raise hospital and medical expense costs in both wealthy and underdeveloped nations, which lowers the standard of healthcare systems. The most common errors that practitioners should exercise great care to avoid are catheter-associated urinary tract infections, central line bloodstream infections, adverse drug events, falls, pressure ulcers, obstetrical adverse events, venous thrombosis, surgical site infections, and the development of ventilator-associated pneumonia. Errors can be prevented by changing the healthcare system to make it more difficult for practitioners to perform incorrect actions and easier for them to do correct ones [6].

More time is spent with patients by nurses than by any other healthcare practitioner, making them the largest profession in the health sector. Therefore, in addition to advocating for patient safety, nurses can significantly reduce errors [7]. Students’ perspectives are how students think to respond about what they have done or about what they learned [8]. The viewpoints of nursing students can shed light on how nursing education helps prepare students to give safe care both while they are enrolled in school and after they become practitioners. Their identification of the strengths and limitations of curriculum and teaching practices can help guide our efforts to enhance nurse education and improve healthcare systems [9].

Therefore, nursing college students must comprehend and develop patient safety competency, as this fosters patients' recuperation, averts unfavorable situations, and has been a global priority for academic and healthcare institutions. Additionally, ensuring patient safety not only improves healthcare outcomes but also enhances the reputation and credibility of healthcare institutions. By prioritizing patient safety, nursing colleges can produce competent and skilled nurses who contribute to the overall development and progress of the healthcare industry [10].

Consequently, we investigated how nursing college final-year students perceived their level of patient safety competency. These results will be useful in formulating plans to raise students' proficiency in patient safety among health professionals.

### Significance of the study

Patient safety issues have become a priority in health policy and healthcare management. It was reported that MEs are the third principal cause of death in the USA, with an estimated 251,000 deaths annually. Patient safety is considered an endemic concern by the WHO. However, literature reports that nursing students might need more knowledge and skills to enhance patient safety. Moreover, the students need help managing errors that might occur [11].

Also, nursing curricula need more emphasis on patient safety. Graduate nurses should have sufficient knowledge to recognize potential safety risks [12]. Sufficient knowledge will increase nursing students' confidence to protect patients from potential harm, errors, and avoidable injuries [13]. Thus, it is imperative to evaluate nursing students’ perspectives on patients' safety competencies.

#### Aim of the study

The study aims to evaluate nursing students’ perspectives on patients' safety competencies.

#### Research objectives

1. Assess nursing students' knowledge regarding patient safety competencies.
2. Evaluate nursing students' perspectives on patient safety competencies.

#### Research questions

What are nursing students’ perspectives on patients' safety competencies?

## Methods

### Research design

A descriptive cross-sectional design was utilized in this study. Descriptive cross-sectional studies explain things or how things are related to each other at a specific time [14]. A descriptive cross-sectional design was suitable for assessing nursing students’ perspectives on patients' safety competencies in accordance with the Strengthening the Reporting of Observational Studies in Epidemiology (STORBE) statement (Appendix).

### Setting

This study was conducted at the Faculty of Nursing, Mansoura University, Egypt.

#### Study sample

A purposive sample of 266 internship nursing students from both genders was included in the study. Purposive sampling was chosen due to its effectiveness in identifying and selecting individuals that meet a predefined set of characteristics essential for the research question. This approach ensured that the participants had a foundational understanding of nursing practices and were in the process of applying these concepts in a clinical environment, making their perspectives on patient safety both unique and immediately relevant. Students were chosen because they have received sufficient training to practice nursing care, and it is also important to investigate nursing safety considerations among these students before offering complete care to patients.

The appropriate sample size for this investigation was determined using the Steven K. Thompson equation [15]. There are 516 students enrolled in nursing internships overall, according to the Student Affairs administration. A minimum of 221 students should be included in the sample size for this study, according to the previously provided data. As the confidence level is 95%, the error proportion is 0.05, and the probabilities are 50%, add 20% for better data and follow-up drop. So the final number should be 266 nursing students.

Inclusion criteria include intern nursing students of both genders who are enrolled in the orientation program in the faculty of nursing at Mansoura University, willing to participate, and signing informed consent. Exclusion criteria include students who have a nursing diploma before joining the faculty of nursing, as those students have more knowledge and clinical experience than other students.

### Tools

One tool was used in this study to collect pertinent data.

#### Patient Safety Survey (PSS)

Our literature review revealed that while there are several established tools for assessing patient safety competencies, most are tailored to qualified healthcare professionals or general nursing students, without a specific focus on internship nursing students in the Egyptian context. Furthermore, our study aimed to explore nuanced aspects of patient safety competencies, including students' perspectives on error reporting and clinical safety issues specific to their internship experiences. These nuances were not adequately covered by existing tools. Therefore, to capture the specific competencies and perspectives of our target population accurately, we decided to develop PSS. Researchers developed this survey after reviewing national and international literature reviews [16–18]. This survey consists of 24 items, divided into two parts. Part one is used to assess internship nursing students’ socio-demographic data. This data includes four items: student name, age, gender, and residence.

Part two is designed to measure internship nursing students’ perspectives regarding patient safety issues. This part covers students’ perspectives in three domains: an overview of patient safety (five items), clinical safety issues (10 items), and error reporting (five items). A 5-point Likert scale, with one representing "strongly disagree" and five representing "strongly agree," was used to gauge the students' perspectives**.**

### Validity and reliability

The researcher developed the study tool after reviewing national and international literature [16–18]. The content validity of the PSS was rigorously evaluated through a structured process involving a panel of seven experts in nursing education, patient safety, and research methodology. These experts were selected based on their extensive experience and contributions to the field, ensuring a comprehensive assessment of the tool's content. Initially, the development of the survey items was informed by an extensive review of both national and international literature on patient safety competencies. This ensured that the content of the tool was grounded in the latest research and best practices in the field. The draft version of the PSS was then presented to the expert panel for evaluation. Each expert independently assessed the relevance, clarity, and comprehensiveness of the survey items, using a standardized scale to rate each item.

Based on the expert ratings, the Content Validity Index (CVI) for the tool was calculated. The CVI provides a quantitative measure of the degree to which experts agree that the survey items are relevant and representative of the construct of patient safety competencies. For our tool, the CVI was calculated at 0.82, indicating a high level of agreement among experts and confirming the content validity of the PSS. A CVI of 0.82 suggests that the majority of the items were deemed relevant and essential for assessing patient safety competencies among nursing students.

In addition to assessing content relevance, the expert panel also provided feedback on the face validity of the tool, focusing on the clarity, simplicity, and readability of the items. This process ensured that the survey would be easily understood by the target population of nursing intern students. Following the expert panel review, several adjustments were made to enhance the clarity and respondent-friendliness of the survey. For instance, the original binary response format was modified to a five-point Likert scale to allow for a more nuanced expression of respondents' perspectives. Additionally, based on expert suggestions, specific items, such as “I know the institution of medicine report, To Error is Human, and its recommendations," were added to enrich the tool's comprehensiveness and relevance. The reliability of the tools was tested using Cronbach’s alpha coefficient (0.89 for the patient safety survey, part two).

#### Pilot study

A pilot study was conducted with 27 participants, representing 10% of the total sample, to test the tool's applicability in the research setting. Feedback from the pilot study identified potential issues and challenges. Modifications were made to the survey tool, ensuring relevance and comprehensibility and addressing practical issues.

### Data collection

Ethical approval was obtained from the Research Ethics Committee of the Faculty of Nursing, Mansoura University**.** The study tool, a patient safety survey, was developed by the researcher based on a recent relevant literature review. A panel of seven experts in the associated fields evaluated the study instrument for face- and content-related validity, and any necessary adjustments were made in response. The reliability of the tools was tested using Cronbach’s alpha coefficient (0.89 for the patient safety survey, part two). A pilot study was carried out with 27 (10%) of the study sample to test the feasibility and applicability of the study tool, and it will be excluded from the study sample. The necessary modifications were made accordingly. The researchers introduced themselves to the selected internship nursing students. The researchers explained the nature and purpose of this study to the study sample. After accepting to participate in this study, the researchers started to collect students’ socio-demographic data and their perspectives regarding patient safety issues using the study tool. Each student was given the appropriate time to answer the patient safety survey (about 20–30 min). The data was collected from January to February 2024.

To avoid bias in the study, we employed a purposive sampling strategy to select a representative sample of internship nursing students from Mansoura University. This strategy was chosen based on specific inclusion and exclusion criteria designed to minimize selection bias and ensure that our sample accurately reflected the population of interest. Additionally, to address potential information bias, we rigorously developed and validated the Patient Safety Survey. The survey underwent a pilot study to identify and correct any ambiguities, further enhancing the reliability and validity of the data collected. The uniform application of a 5-point Likert scale across all survey items was a deliberate choice to provide a consistent measure of nursing students' perspectives, thereby reducing measurement bias. Additionally, we standardized the training for all researchers involved in data collection to ensure uniform survey administration. We took several measures to minimize response bias, including guaranteeing anonymity and confidentiality for all participants and making participation entirely voluntary. These steps were intended to foster an environment where students felt comfortable providing honest and accurate responses without fear of repercussions.

### Statistical analysis of the data

The computer was fed data, and IBM SPSS software package version 20.0 was used for analysis. [IBM Corp. Armonk, NY] Numbers and percentages were used to describe the qualitative data. The distribution's normality was confirmed using the Kolmogorov–Smirnov test. The range (minimum and maximum), mean, standard deviation, and median were used to characterize quantitative data. The results were deemed significant at the 5% level. Student t-test: to compare two examined categories for quantitative variables that are regularly distributed. F-test (ANOVA): for normally distributed quantitative variables, to compare between more than two categories. Pearson coefficient: to correlate between two normally distributed quantitative variables.

### Ethical considerations and human rights

The Research Ethical Committee of the Faculty of Nursing at Mansoura University in Egypt provided ethical permission (No.0526). After being fully informed about the purpose of the study, each intern nursing student who was enrolled gave their informed consent. The pupils were reminded by the researcher that participation is completely voluntary. Throughout the whole study, confidentiality, privacy, safety, and anonymity were guaranteed. Every participant was free to leave the research at any moment. The survey did not include participant names or any other type of identifying information. The Helsinki Declaration and other pertinent rules and regulations carry out every procedure.

## Results

### Demographic characteristics

The study included a total of 266 students. About 57.9% of the involved students were aged 22, and 65% of them were female. Moreover, 64.7% of the enrolled students lived in rural areas. All the involved students (100%) were from Mansoura University (Table 1).

**Table 1:** Distribution of the studied students according to demographic data (*n* = 266)

Demographic data	No	%
**Age in years**		
21	17	6.4
22	154	57.9
23	95	35.7
**Min. – Max**	21.0 – 23.0	
**Mean ± SD**	22.29 ± 0.58	
**Median**	22.0	
**Gender**		
Male	93	35.0
Female	173	65.0
**Residence**		
Rural	172	64.7
Urban	94	35.3
**University name (Mans)**	266	100.0

*SD* Standard deviation

### Students’ distribution according to the patient safety overview domain

Among the studied intern nursing students, we found that 55.3%, 59.4%, 40.6%, 41.7%, and 49.6% of the involved students agreed that they can understand the concept of patient safety, understand the burden of medical errors, differentiate between errors, adverse events, close call/near miss, and sentinel events, know the Institution of Medicine report “To Error is Human” and its recommendations, and are aware of the ethical aspect of patient safety. The total score of the patient safety overview domain (mean ± SD) was 19.76 ± 2.69 (Table 2).

**Table 2:** Distribution of the studied students according to the patient safety overview domain (*n *= 266)

Patient safety overview	Strongly disagree		Disagree		Neutral		Agree		Strongly agree		Mean ± SD	Median
	**No**	**%**	**No**	**%**	**No**	**%**	**No**	**%**	**No**	**%**		
I understand the concept of patient safety	2	0.8	1	0.4	27	10.2	147	55.3	89	33.5	4.20 ± 0.69	4.0
I understand the burden of medical errors	2	0.8	7	2.6	39	14.7	158	59.4	60	22.6	4.0 ± 0.74	4.0
I can differentiate between error, adverse event, close call/near miss, and sentinel event	2	0.8	21	7.9	75	28.2	108	40.6	60	22.6	3.76 ± 0.92	4.0
I know the institution of medicine report To Error is human, and its recommendations	3	1.1	25	9.4	70	26.3	111	41.7	57	21.4	3.73 ± 0.94	4.0
I am aware of the ethical aspect of patient safety	3	1.1	5	1.9	45	16.9	132	49.6	81	30.5	4.06 ± 0.81	4.0
Patient safety Overview total score	Min. – Max						12.0 – 25.0					
	Mean ± SD						19.76 ± 2.69					
	Median						20.0					

### Distribution of the studied students according to clinical safety issues

Regarding clinical safety issues, 50.4%, 51.1%, 54.9%, 52.3%, and 52.3% of the participating students agreed that they felt confident in what they had learned about curbing infection spread, identifying patients correctly, avoiding surgical errors, using medicines safely, and preventing venous thromboembolism, respectively. In addition, 51.1%, 52.3%, 47.7%, 48.1%, and 48.5% of the participating students agreed that they felt confident in what they had learned about customizing hospital discharges, using good hospital design principles, assembling better teams and rapid response systems, sharing data for quality improvement, and fostering an open-communication culture (Table 3).

**Table 3:** Distribution of the studied students according to clinical safety issues (*n* = 266)

Clinical safety issues	Strongly disagree		Disagree		Neutral		Agree		Strongly agree		Mean ± SD	Median
	No	%	No	%	No	%	No	%	No	%		
I feel confident in what I learned about…												
Curbing infection spread	5	1.9	6	2.3	62	23.3	134	50.4	59	22.2	3.89 ± 0.84	4.0
Identifying patients correctly	2	0.8	4	1.5	41	15.4	136	51.1	83	31.2	4.11 ± 0.76	4.0
Avoiding surgical errors	6	2.3	9	3.4	48	18.0	146	54.9	57	21.4	3.90 ± 0.85	4.0
Using medicines safely	4	1.5	5	1.9	41	15.4	139	52.3	77	28.9	4.05 ± 0.81	4.0
Preventing venous thromboembolism	6	2.3	6	2.3	57	21.4	139	52.3	58	21.8	3.89 ± 0.85	4.0
Customizing hospital discharges	3	1.1	15	5.6	71	26.7	136	51.1	41	15.4	3.74 ± 0.83	4.0
Using good hospital design principles	0	0.0	18	6.8	66	24.8	139	52.3	43	16.2	3.78 ± 0.80	4.0
Assembling better teams and rapid response systems	2	0.8	14	5.3	78	29.3	127	47.7	45	16.9	3.75 ± 0.82	4.0
Sharing data for quality improvement	0	0.0	17	6.4	55	20.7	128	48.1	66	24.8	3.91 ± 0.84	4.0
Fostering an open-communication culture	2	0.8	14	5.3	57	21.4	129	48.5	64	24.1	3.90 ± 0.85	4.0
Clinical safety issues total score	Min. – Max						19.0 – 50.0					
	Mean ± SD						38.91 ± 5.15					
	Median						39.0					

### Distribution of the studied students according to error reporting issues domain

Concerning their error reporting, 40.2%, 50%, 37.2%, 44.7%, and 41% of the involved students agreed that they were aware of error reports, understood the importance of incident reports, enumerated the barriers to incident reporting, listed the features of an incident report, and differentiated between manual and electronic incidence reports (Table 4).

**Table 4:** Distribution of the studied students according to error reporting issues domain (*n* = 266)

Error reporting issues	Strongly disagree		Disagree		Neutral		Agree		Strongly agree		Mean ± SD	Median
	No	%	No	%	No	%	No	%	No	%		
I am aware of error reports	4	1.5	12	4.5	88	33.1	107	40.2	55	20.7	3.74 ± 0.89	4.0
I understand the importance of the incident report	1	0.4	13	4.9	65	24.4	133	50.0	54	20.3	3.85 ± 0.81	4.0
I can numerate the barriers to incident reporting	10	3.8	27	10.2	86	32.3	99	37.2	44	16.5	3.53 ± 1.01	4.0
I can list the features of an incident report	4	1.5	30	11.3	69	25.9	119	44.7	44	16.5	3.64 ± 0.94	4.0
I can differentiate between manual and electronic incidence report	12	4.5	22	8.3	71	26.7	109	41.0	52	19.5	3.63 ± 1.03	4.0
Error reporting issues total score	Min. – Max						8.0 – 25.0					
	Mean ± SD						18.38 ± 3.47					
	Median						18.50					

### Relation between nursing students’ perspectives toward patient safety, their gender, and their age

Regarding gender, there was no statistically significant difference between nursing students' perceptions of patient safety and their gender (*p* > 0.05). At the same time, there was a statistically significant difference between the nursing student patient safety overview domain and their age (*p* = 0.025) (Table 5).

**Table 5:** Relation between nursing students’ perspectives toward patient safety, their gender, and their age (*n* = 266)

Total Score	Gender		t	P	Age in years			F	p
	Male (*n* = 93)	Female (*n* = 173)			21 (*n* = 17)	22 (*n* = 154)	23 (*n* = 95)		
	Mean ± SD	Mean ± SD			Mean ± SD	Mean ± SD	Mean ± SD		
Patient safety overview	20.17 ± 2.64	19.54 ± 2.70	1.825	0.069	21.18 ± 2.70	19.87 ± 2.64	19.34 ± 2.70	3.736	0.025^*^
Clinical safety issues	38.91 ± 4.83	38.91 ± 5.33	0.001	0.999	40.88 ± 4.91	38.81 ± 5.60	38.74 ± 4.37	1.333	0.265
Error reporting	18.05 ± 3.39	18.55 ± 3.51	1.123	0.262	19.47 ± 3.81	18.27 ± 3.35	18.36 ± 3.61	0.913	0.402
Overall Patient safety	77.14 ± 9.11	77.01 ± 10.32	0.101	0.920	81.53 ± 9.82	76.95 ± 10.26	76.43 ± 9.17	1.950	0.144

t: Student t-test

p: *p*-value for comparison between the studied categories

F: F for One-way ANOVA test

^*^: Statistically significant at *p* ≤ 0.05

### Correlation among nursing students’ perspectives domains toward patient safety

There were very high positive correlations between the overall patient safety score and its three domains: the patient safety overview domain (*r* = 0.806, *p* < 0.001), the clinical safety issues domain (*r* = 0.932, *p* < 0.001), and the error reporting domain (*r* = 0.842, *p* < 0.001). Moreover, there was a statistically significant difference between the patient safety overview domain and the clinical safety issues domain (*p* < 0.001) with a high positive correlation (*r* = 0.659). In addition, there was a moderately positive correlation between the patient safety overview domain and the error reporting domain with a statistically significant difference (*r* = 0.543, *p* < 0.001). Also, there was a high positive correlation between the clinical safety issues domain and the error reporting domain (*r* = 0.660, *p* < 0.001) (Table 6).

**Table 6:** Matrix correlation among nursing students’ perspectives domains toward patient safety

Items		Patient safety overview	Clinical safety issues	Error reporting	Overall patient safety
Patient safety overview	**R**	1.000	0.659	0.543	0.806
	**P**		< 0.001^*^	< 0.001^*^	< 0.001^*^
Clinical safety issues	**R**		1.000	0.660	0.932
	**P**			< 0.001^*^	< 0.001^*^
Error reporting	**R**			1.000	0.842
	**P**				< 0.001^*^
Overall Patient safety	**R**				1.000
	**P**				

r: Pearson coefficient

^*^: Statistically significant at *p* ≤ 0.05

## Discussion

Nursing students are the foundation upon which nursing care for patients will be built, and patient safety must be considered the cornerstone of the student’s education before graduation to prepare them well to provide the best care with the highest quality and efficiency [19]. Working across professions in clinical fields requires an early understanding of the responsibilities of different healthcare providers and the extent of nursing students' engagement [20].

Using a self-reported approach, we evaluated nursing students' perspectives of patient safety competency concerning safety overview, clinical safety issues, and error reporting issues. Our study's compelling data demonstrated that intern students who took part in the patient safety survey scored higher overall in all patient safety-related categories. When it came to clinical safety considerations, the students received the highest percentage of points. On the other hand, problems with error reporting showed the lowest percentage.

The clinical safety dimension, with its focus primarily on infection control, patient identification, safe medication administration, and waste disposal, might be the most familiar to students, as our students start clinical training from the first academic level in the hospital with regular and varied evaluations that help them to have a comprehensive understanding of nursing students' proficiency in infection control and patient identification. Another possible explanation for this is that combining written assessments, practical evaluations, simulations, and real-world clinical experiences in our faculty allows educators to gauge students' competence and readiness for professional practice, which increases their knowledge base.

This is in line with the results of a study in Portugal, which reported a high perception of students in terms of infection control [21]. Another study conducted in Saudi Arabia indicated a modest perception among nursing students regarding infection prevention [22]. Regarding the error reporting issue, this is because students were worried about disciplinary actions, damage to their reputation, or a potential impact on their academic and professional future. Also, the majority of our students are from rural areas with a blame culture present that can discourage open communication about error reporting.

Another significant aspect of the safety overview domain is that students have a deeper perspective on the burden of medication errors and the concept of patient safety. This finding might relate to prior exposure to patient safety-related topics. This is in harmony with those of Chan 2019, who reported students had a good perception of general terms and the concept of safety [23]. Another study assessing medical students’ knowledge, skills, and attitudes also reported high perceptions of students regarding general aspects of patient safety [24].

Another interesting finding regarding clinical safety issues is that the high perspective and confidence percentage about avoiding surgical error and the lowered perspective percentage represented assembling better teams and rapid response systems. We attribute this superiority in preventing surgical errors to the fact that the majority of respondents work part-time in the surgical and plastic surgery hospitals spread across the governorate, which gave them practical experience in this part. In combination with education, experience, mentorship, and a supportive healthcare culture, this contributes to nursing interns developing a positive perception regarding avoiding surgical errors. Following the present results, a previous study in Turkey demonstrated that nurses who formerly received preparation on patient safety had a higher statistical percentage [25]. However, the findings of the current study do not support the previous research that reported that pre-licensure nursing students have little knowledge regarding perioperative care and should be well-trained again [26].

Regarding lack of perspective in assembling a better team and rapid response system, because interns feel hesitant to voice concerns or take charge due to hierarchical structures, insufficient resources, both in terms of staffing and equipment, may hinder the interns' ability to assemble an effective team and respond. This outcome is contrary to that of Kamran, who reported that the best score of safety was given for team functioning and response [27].

Regarding gender, there was no statistically significant difference between nursing students’ perspectives on patient safety and their gender (*p* > 0.05). This is in line with those of Ramírez, who reported that the differences in means between genders were not significantly different in the overall perspective of patient safety [28]. Additionally, those who stated that there were no discernible variations in opinions about gender and past exposure to medical errors (*p* =  > 0.05) [27]. This outcome is contrary to that stated: male students apparent competence in “working in teams” is higher than that of females [29].

Another pilot study reported that the overall patient safety grade, the number of reported events, and the number of reported events by nursing students were significantly predicted by several patient safety competence dimensions (*p* ≤ 0.05) [30].

Our results indicated that there is a significant relationship between age and patient safety. The rationale of this finding is that during the academic years, including clinical practicum, students’ ability to communicate with patients and other health professionals clearly and consistently seemed to increase with age. Similar positive student assessments about safety and age have been noted in a study by Usher, who reported highly significant scores of patient safety with age and level of students. The results are also inconsistent with those conducted in Australia and New Zealand that assess nursing students' patient safety knowledge. These results corroborate the findings of a great deal of the previous work reported a significant difference was found in the patient safety competence of nursing students with year of study [29].

Another finding that stands out from the results is that there were very high positive correlations between the overall patient safety score and the three domains. These results reflect those of another study that examined the relationship between all-cause harm and patient safety and demonstrated strong correlations between all-cause harm measures and patient safety culture [31]. These findings also lend support to previous literature, which reported that subscales of safety correlated positively with the perceived patient safety culture scale [32]. Our finding also supports evidence from previous observations that found a positive correlation between the six domains and safety-related behaviors [33].

Another finding is that there was a statistically significant difference between the patient safety overview domain and the clinical safety issues domain. The same results were reported in a cross-sectional study conducted in China that assessed the patient safety competency of Chinese nurses [34]. Also, there was a high positive correlation between the clinical safety issues domain and the error reporting domain; this finding is consistent with Mahsoon [35]. This finding is contrary to the findings of another Saudi cross-sectional study that showed a significant negative correlation [36]. Another vital aspect of patient safety that students recognized is likewise related to understanding the function of trust and error reporting in maintaining patient safety.

## Conclusion

Nursing students ought to have a strong understanding of patient safety, grounded in the highest standards of nursing care. Students completing nursing internships knew about patient safety. This result supports the conclusion drawn from several recent studies that patient safety education improves nurses' patient safety competence. These elements could have an impact on nursing students' patient safety competence and performance. The intern students would benefit from additional educational and training workshops to increase their perspectives on patients' safety competencies. Therefore, we recommend that academic institutions and medical facilities reorganize the framework for patient safety education to begin at the earliest academic level while taking into account students' pedagogical demands and varying safety levels. This will be done to increase public awareness of patient safety education. Establishing a structured curriculum on patient safety and upholding this shift in hospital culture is also crucial if we are to optimize the impact of patient safety education. Future research in various cultural and contextual settings is necessary to enhance our understanding of the variables affecting patient safety in nursing practice and education.

### Limitations

When evaluating the results, it is important to take into account the study's limitations, which include its cross-sectional design and the inclusion of only one site. An additional constraint pertains to the survey's timing, which was carried out during the internship's orientation program. The student was not entirely tasked with providing comprehensive and intense care to patients with minimal exposure to clinical safety and real-error reporting concerns. The results could have been altered if the data had been gathered closer to the internship's conclusion, when the students would have gained more clinical experience. The study was conducted at a single nursing faculty; the use of purposive sampling, while ensuring a detailed exploration of our specific research question, may also limit the generalizability of the results. Therefore, it is recommended that it be repeated across other faculties to enable generalization of results.

### Supplementary Information

**Supplementary Material 1.**

## Data Availability

The datasets generated and/or analyzed during the current study are not publicly available due to protecting the confidentiality of the participants, but are available from the corresponding author upon reasonable request.
