# Supplementary material for: Nursing students’ perspectives on patients' safety competencies: a cross-sectional survey
Source: BMC Nurs. 2024 May 13;23:323. doi: 10.1186/s12912-024-01966-1 (PMC11089785; doi:10.1186/s12912-024-01966-1)
Supplement: Supplementary file 1 — Supplementary Material 1. [file 12912_2024_1966_MOESM1_ESM.doc]

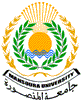

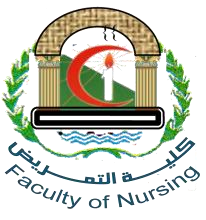


**Nursing students’ perspectives on patients' safety competencies: A cross-sectional survey**

**Dear student:**

All of these data will be involved in this form will remain confidential and will be use in scientific research purpose only.

| **Structure Interview Sheet** | | | | |
| --- | --- | --- | --- | --- |
| **Part one; a demographic data** | | | | |
|  | | | | student name |
|  |  |  |  | Age in years |
|  |  |  |  |
|  |  |  |  |
|  |  |  |  |
|  | Female |  | Male | Gender |
|  | Urban |  | Rural | Residence |

| **Part two; patient safety survey/ scale** | | | | | |
| --- | --- | --- | --- | --- | --- |
| **Domain** | **Strongly disagree** | **Disagree** | **Neutral** | **Agree** | **Strongly Agree** |
| **Patient safety overview** | | | | | |
| 1. **I understand the concept of patient safety** |  |  |  |  |  |
| 1. **I understand the burden of medical errors** |  |  |  |  |  |
| 1. **I can differentiate between error, adverse event, close call/near miss, and sentinel event** |  |  |  |  |  |
| 1. **I know Institution of medicine report To Error is human, and its recommendations** |  |  |  |  |  |
| 1. **I aware about Ethical aspect of patient safety** |  |  |  |  |  |
| **Clinical safety issues** | | | | | |
| 1. **I feel confident in what I learned about curbing infection spread** |  |  |  |  |  |
| 1. **I feel confident in what I learned about identifying patients correctly** |  |  |  |  |  |
| 1. **I feel confident in what I learned about avoiding surgical errors** |  |  |  |  |  |
| 1. **I feel confident in what I learned about using medicines safely** |  |  |  |  |  |
| 1. **I feel confident in what I learned about preventing venous thromboembolism (VTE)** |  |  |  |  |  |
| 1. **I feel confident in what I learned about customizing hospital discharges** |  |  |  |  |  |
| 1. **I feel confident in what I learned about using good hospital design principles** |  |  |  |  |  |
| 1. **I feel confident in what I learned about assembling better teams and rapid response systems** |  |  |  |  |  |
| 1. **I feel confident in what I learned about sharing data for quality improvement** |  |  |  |  |  |
| 1. **I feel confident in what I learned about fostering an open-communication culture** |  |  |  |  |  |
| **Error reporting** | | | | | |
| 1. **I aware about error reports** |  |  |  |  |  |
| 1. **I understand the importance of incidents report** |  |  |  |  |  |
| 1. **I can numerate the barriers to incident reporting** |  |  |  |  |  |
| 1. **I can list the features of an incident report** |  |  |  |  |  |
| 1. **I can differentiate between manual and electronic incidence report** |  |  |  |  |  |
